# Extracellular matrix remodelling in obesity and metabolic disorders

**DOI:** 10.1093/lifemeta/load021

**Published:** 2023-05-26

**Authors:** Vishal Musale, David H Wasserman, Li Kang

**Affiliations:** Division of Systems Medicine, School of Medicine, University of Dundee, Dundee DD1 9SY, United Kingdom; Department of Molecular Physiology and Biophysics, Mouse Metabolic Phenotyping Center, Vanderbilt University, Nashville, TN 37235, United States; Division of Systems Medicine, School of Medicine, University of Dundee, Dundee DD1 9SY, United Kingdom

**Keywords:** extracellular matrix, fibrosis, insulin resistance, obesity, metabolism

## Abstract

Obesity causes extracellular matrix (ECM) remodelling which can develop into serious pathology and fibrosis, having metabolic effects in insulin-sensitive tissues. The ECM components may be increased in response to overnutrition. This review will focus on specific obesity-associated molecular and pathophysiological mechanisms of ECM remodelling and the impact of specific interactions on tissue metabolism. In obesity, a complex network of signalling molecules such as cytokines and growth factors has been implicated in fibrosis. Increased ECM deposition contributes to the pathogenesis of insulin resistance at least in part through the activation of cell surface integrin receptors and CD44 signalling cascades. These cell surface receptors transmit signals to the cell adhesome which orchestrates an intracellular response that adapts to the extracellular environment. Matrix proteins, glycoproteins, and polysaccharides interact through ligand-specific cell surface receptors that interact with the cytosolic adhesion proteins to elicit specific actions. Cell adhesion proteins may have catalytic activity or serve as scaffolds. The vast number of cell surface receptors and the complexity of the cell adhesome have made study of their roles challenging in health and disease. Further complicating the role of ECM-cell receptor interactions is the variation between cell types. This review will focus on recent insights gained from studies of two highly conserved, ubiquitous axes and how they contribute to insulin resistance and metabolic dysfunction in obesity. These are the collagen-integrin receptor-IPP (ILK-PINCH-Parvin) axis and the hyaluronan-CD44 interaction. We speculate that targeting ECM components or their receptor-mediated cell signalling may provide novel insights into the treatment of obesity-associated cardiometabolic complications.

## Overview of extracellular matrix (ECM) remodelling in obesity and insulin resistance

The ECM is a dynamic network of proteins, proteoglycans, polysaccharides, and biologically active factors that provide structural support and information pertaining to the status of the extracellular environment to cells [1]. The ECM remodels as a protective mechanism in response to inflammation or injury. The ECM also expands in response to obesity resulting from habitual excesses of calorie intake. This is neither a corrective nor protective response. Never over the course of human evolution was there the selective pressure to adapt to chronic overnutrition. The consequence is that the ECM remodelling associated with obesity may be maladaptive, resulting in the accumulation of ECM molecules and the activation of ECM membrane receptors (e.g. integrins and CD44) [2–8]. ECM activation of cell surface receptors has been implicated in the pathogenesis of a spectrum of cardiometabolic diseases [9–11]. The ECM as a mechanism of tissue dynamic remodelling shows diverse profiles and executes distinct regulatory processes in different metabolic tissues as reviewed previously [12].

The ECM is classified into two types based on location: interstitial (e.g. collagens I, III, and V) and basement membrane (e.g. collagen IV, laminin, fibronectin, and hyaluronan) matrix. Interstitial ECM components are primarily produced by mesenchymal cells including fibroblasts and myofibroblasts. These cells are believed to regulate ECM homeostasis by synthesizing, degrading, and organizing ECM components [13, 14]. Proteins of the basement membrane are produced by epithelial cells, endothelial cells, and pericytes, and represent tissue specificity [15]. The ECM influences a range of cellular processes, such as cell proliferation, differentiation, and migration [16], via interacting with cell surface receptor integrins [17]. These glycoprotein receptors are αβ heterodimers with extracellular, single transmembrane, and cytoplasmic domains. Integrins have been demonstrated to influence biological activity by sending signals bi-directionally across the cell membrane [18, 19]. In addition to its role in cellular processes, the ECM also serves as a reservoir of growth factors. These include, but are not limited to, transforming growth factor-β (TGF-β), fibroblast growth factor, and vascular endothelial growth factor (VEGF).

ECM components undergo dynamic changes in the deposition and composition and are important in the preservation of normal tissue function [19, 20], as well as during inflammation and for wound healing and tissue regeneration that may occur with tissue injury [12, 21]. Such remodelling is regulated under physiological settings by a delicate balance of synthesis, post-translational modifications, and degradation of its constituents [21]. Studies with selective deletion of ECM components demonstrate the importance of the ECM in tissue homeostasis by exerting control of cellular senescence, proliferation, migration, and differentiation [22]. The change in the extracellular environment resulting from ECM expansion with obesity provokes an adaptive response by the cell which may contribute to impaired tissue function and lead to disease development.

ECM remodelling in disease states or with obesity is initially invoked to retain the structural and functional features of the organ, but a persistent expansion of the ECM may evolve into maladaptive fibrosis [23]. Pathological ECM remodelling can be triggered by hypoxia, inflammation, biomechanical stress, and excessive neurohormonal activation. Increased ECM causes tissue stiffness and organ dysfunction, which is exacerbated by increased cross-linking of ECM components [24]. Abnormal deposition of ECM components is evident in clinical conditions such as lung fibrosis, liver cirrhosis, and cardiovascular diseases [16].

Obesity is a risk factor for a cluster of chronic illnesses including insulin resistance, diabetes, heart disease, fatty liver disease, and atherosclerosis. The insulin resistance of obesity is an independent risk factor that may contribute to the pathophysiological relationship between obesity and associated metabolic disorders [25]. During the progression of obesity, the ECM expands dynamically in metabolic tissues [26–28]. Evidence from both human and rodent suggests that pathological ECM remodelling contributes to insulin resistance in obesity. Individuals with obesity and insulin resistance display increased ECM deposition in adipose tissue compared to those with obesity of equivalent BMI and normal insulin sensitivity, implying that abnormal ECM remodelling is crucial in the pathophysiology of insulin resistance [29]. Rapid weight gain in healthy people results in muscle insulin resistance and upregulation of muscle ECM genes [30]. Moreover, in the insulin-resistant muscles of patients with obesity and Type 2 diabetes, collagen expression (i.e. collagens I and III) is increased [31, 32]. The beneficial effects of exercise on muscle insulin sensitivity in patients post-bariatric surgery are partially related to a decrease in muscle collagens I and III expressions [33]. Furthermore, a genome-wide epigenetic investigation of adipose tissues of individuals with obesity and insulin resistance shows novel insulin resistance-related genes that pertain to ECM and its interaction with the cell. These include *COL9A1*, *COL11A2*, and *CD44* [34].

The underlying factors for obesity-induced ECM remodelling are unknown. The inflammatory response associated with obesity is considered a predominant mechanism. Adipose tissue, the primary site where inflammation is initiated and exacerbated in obesity, has been the subject of numerous studies [35–37]. Excess nutrients cause adipocytes to enlarge and proliferate, which in turn induces hypoxia, mechanical stress, and cell death; these signals trigger inflammation, which is manifested by an increase in inflammatory cytokines and TGF-β [37, 38]. The production and secretion of several inflammatory mediators increase the infiltration of monocytes in adipose tissue and promote their differentiation to proinflammatory macrophages which produce and secret many more proinflammatory mediators that eventually trigger local and systemic inflammation [39, 40]. Inflammation causes the fibrogenic response resulting in increased production and accumulation of ECM proteins. An alternative sequalae has also been proposed in the adipose tissue where fibrosis induces inflammation [41]. This will be further discussed later in the review.

**Figure 1:**
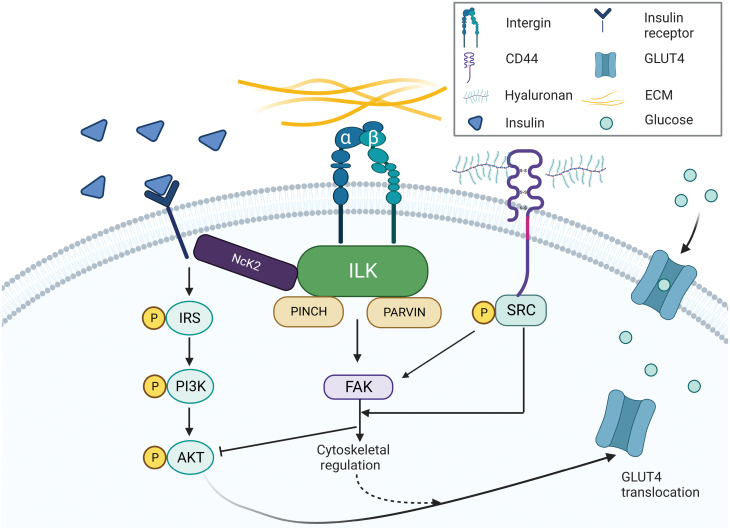
Pathological ECM remodelling in obesity. Obesity induces a maladaptive ECM remodelling by increasing deposition of its components and activating downstream ECM receptor signalling (e.g. integrin and CD44). ILK, a primary modulator of integrin signalling, interacts with the cytoplasmic domain of integrins and forms an ILK-PINCH-Parvin (IPP) protein complex, which recruits adaptor proteins such as Nck2 to interact with tyrosine kinase receptors including the insulin receptor. Under obese condition, overactivation of CD44 signalling has been shown to suppress AKT phosphorylation. In response to excessive ECM deposition in obese state, integrin and CD44 signalling cascades have been linked to impaired GLUT4 translocation and glucose transport under insulin stimulation in fat and muscle cells.

Several lines of evidence suggest that ECM expansion and activation of its downstream ECM receptor signalling are linked to insulin resistance in diet-induced obesity (Fig. 1). Recent studies in skeletal muscle, adipose tissue, and liver explored the association between obesity-induced ECM remodelling, integrin signalling, and insulin resistance. Tissue-specific deletion of integrin-linked kinase (ILK) (Box 1), an intracellular adaptor protein of integrin receptor signalling, in skeletal muscle, liver, and adipose tissue ameliorates high fat diet-induced tissue-specific insulin resistance [6, 8, 42]. Moreover, CD44 (Box 1), one of the main cell surface receptors for the ECM hyaluronan, is implicated in obesity and Type 2 diabetes, as genetic ablation of *CD44* or its pharmacological inhibition improves diet-induced disruptions of glucose homeostasis in mice [7, 43, 44]. High fat diet feeding in mice increases CD44 protein expression in muscle, and mice lacking *CD44* gene have increased muscle vascularization and ameliorate diet-induced insulin resistance in skeletal muscle [7]. These findings suggest that obesity-driven ECM remodelling/deposition and activation of its downstream signalling are necessary for obesity-induced insulin resistance.

## Molecular pathophysiological mechanisms of ECM remodelling in obesity and insulin resistance

**Figure 2:**
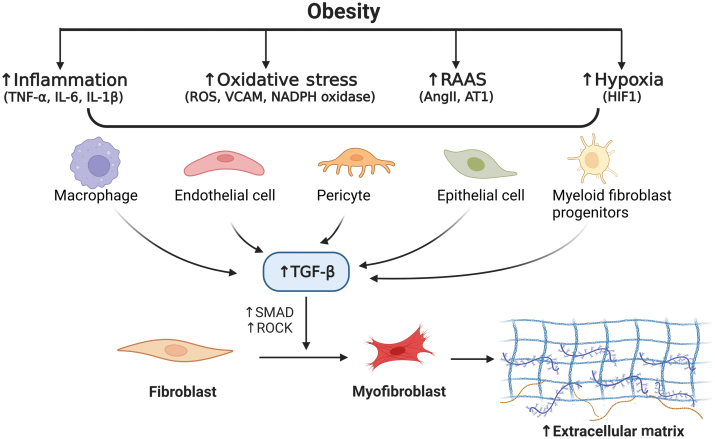
Potential mechanisms of pathological ECM remodelling in obesity. The molecular and pathophysiological mechanisms that underly maladaptive ECM remodelling in obesity are depicted. Obesity-related inflammation, oxidative stress, RAAS, and hypoxia have been shown to stimulate and activate ECM-producing fibroblast cells, promoting matrix synthesis. Obesity induces inflammation with elevated levels of TNF-α, IL-1β, and IL-6. Oxidative stress in obesity is manifested by increases in ROS levels. Activation of RAAS also leads to increases in ROS via inducing the expression of NADPH oxidase. The expression of vascular cell adhesive molecule (VCAM) is upregulated by ROS and HIF1, which is induced in response to hypoxia. These signals could increase TGF-β expression and initiate TGF-β mediated profibrotic response in various cells including macrophages and endothelial cells. TGF-β has been identified as an important regulator of maladaptive ECM remodelling, promoting excess deposition of ECM components possibly through activation of signalling molecules like SMAD and Rho-associated protein kinase (ROCK).

The molecular pathophysiology of obesity-driven ECM remodelling has been extensively studied and attributed to inflammation, hypoxia, renin-angiotensin-aldosterone system (RAAS), TGF-β signalling, and oxidative stress (Fig. 2).

### Inflammation

Inflammation can cause pathological ECM remodelling or fibrosis through immune system activation in obesity [45]. Immune cells can produce structural ECM proteins upon activation and polarization or act as a key effector by synthesizing fibrogenic mediators [46–48]. Proinflammatory cytokines such as tumour necrosis factor alpha (TNF-α), interleukin-1 (IL-1), and IL-6 have been linked to fibrosis either through a direct effect on ECM-producing fibroblast cells or by promoting immune cell infiltration and activation [49, 50]. In skeletal muscle, increased collagen deposition is associated with increased gene expression of TNF-α and F4/80, a macrophage infiltration marker, in obesity and insulin resistance [2]. In contrast, when collagen deposition is normalized by either reduction of mitochondrial oxidative stress or inhibition of phosphodiesterase (PDE) 5a, expression of TNF-α and F4/80 in skeletal muscle is normalized and insulin resistance in mice is improved [2]. In the liver, increased expression of osteopontin, an ECM glycoprotein that plays a vital role in the development of hepatic steatosis and insulin resistance, is linked to increased expression of TNF-α and the macrophage markers F4/80 and CD68 in the liver as well as abdominal subcutaneous adipose tissue in obesity [51, 52]. Moreover, excess myocardial collagen deposition is linked to high circulating levels of IL-6 and TNF-α in patients with obesity and heart failure [53, 54]. Elevated numbers of inflammatory cells, including CD3, CD11a, and CD45 positive cells, are also positively correlated with collagen deposition in the cardiac tissue of patients with heart failure with preserved ejection fraction (HFpEF) [55]. In high fat diet-fed hypertensive rats, elevated expression of TNF-α in cardiac tissue is associated with a marked increase in collagen deposition, which contributes to impaired ventricular function [56].

Moreover, studies in mice have implicated a causative role of TNF-α in the pathogenesis of fibrosis. Blocking TNF-receptor-1 (TNFR1) with an anti-TNFR1 antibody reduces liver fibrosis and steatosis in high fat-fed obese mice [57]. Loss of TNFR1 in hepatic stellate cells (HSC) reduces pro-collagen-α1(I) mRNA expression and decreases HSC proliferation [58]. These results were further validated in human HSC cell lines and TNFR1 knockout mice [58]. By contrast, transgenic mice with cardiac-specific overexpression of TNF-α develop progressive ventricular hypertrophy and dilation, which is accompanied by an increase in matrix metalloproteinase (MMP)-2 activity, and collagen synthesis and deposition [59]. In addition to TNF-α, IL-6 stimulation increases cardiac fibroblast proliferation and collagen production, whereas knocking out IL-6 reduces cardiac fibroblast collagen production in response to high glucose stimulation [60]. In streptozotocin-induced diabetic mice, IL-6 deletion improves cardiac function and reduces interstitial fibrosis [60].

Obesity causes chronic low-grade inflammation in adipose tissue which has been attributed to mechanical stress of adipocyte expansion, hypoxia, and subsequent immune and mast cell infiltration, which contributes to the production of excess ECM components from a variety of cells including adipose stem and progenitor cells, adipocytes, fibroblasts, immune cells, and mast cells. Adipose tissue fibrosis and inflammation during obesity have been extensively reviewed recently [41, 61]. Hypoxia has been recognized as a key initiating step in the obese adipose tissue to induce inflammation and fibrosis [41]. This is discussed in the following section. Apart from the concept that inflammation leads to fibrosis, it is worth noting that fibrosis can also induce inflammation by increasing the mechanical stress on adipocytes from tissue stiffness [45], although the detailed molecular signals are not fully understood.

### Hypoxia

Hypoxia causes aberrant adipose tissue ECM remodelling by modulating the biosynthesis of fibril collagens, the expression of intracellular collagen-modifying enzymes, and ECM degradation via hypoxia-inducible factors (HIFs). As adipocytes grow larger due to lipid accumulation in response to overnutrition, oxygen delivery becomes limiting, resulting in hypoxia [35, 62, 63]. Hypoxia stimulates pathological ECM protein remodelling, stress signals, and angiogenesis via the expression of HIF1. Increased expression of HIF1 has been linked to increased macrophage infiltration and expression of genes involved in angiogenesis, collagen synthesis, and biosynthetic enzymes like lysyl oxidase (LOX) [62, 64, 65]. Increased collagen deposition inhibits the physiological dynamics of the ECM required by healthy adipocytes, resulting in lipid deposition in ectopic depots in tissues such as liver, skeletal muscle, pancreas, and heart [66]. Lipid metabolites promote local inflammation and insulin resistance [67]. In addition, hypoxia activates proangiogenic factors such as VEGF and platelet-derived growth factor (PDGF), which are produced by both adipocytes and adipocyte progenitor cells [68]. Despite the compensatory activation of proangiogenic factors, obesity is associated with decreased vascular density due to endothelial dysfunction and a counteracting increase in anti-angiogenic molecules [69]. Indeed, loss of VEGF shifts the balance of proangiogenic to anti-angiogenic response, resulting in reduced formation of blood vessels and excess collagen deposition and fibrosis, which is marked by increased HIF expression [70]. Therefore, VEGF expression in adipose tissue may reverse obesity-induced adverse ECM remodelling by promoting angiogenesis preventing adipocyte hypoxia and impaired lipid storage [71–73]. While hypoxia as a driver of fibrosis is most relevant in tissues such as adipose tissue in obesity, where capillary perfusion and oxygen delivery become limiting oxygen pressure (pO_2_), it may be less critical in organs that remain well-perfused despite obesity such as the liver [74]. Thus, the role of hypoxia in initiating pathological ECM remodelling outside adipose tissue remains to be further investigated.

### RAAS

RAAS is critical to the regulation of blood pressure and electrolyte balance homeostasis. Activation of the RAAS system has been linked to the cardiometabolic pathology associated with obesity [75]. Components of the RAAS system have been shown to induce a profibrotic response by activating ECM-producing fibroblast cells, resulting in their proliferation and induction of matrix-synthetic, and preserving pathways [27]. Angiotensin II (AngII) is known to stimulate a variety of fibrogenic actions of fibroblasts, including cell migration [76], proliferation [77], proinflammatory cytokine secretion, and collagen synthesis [78], primarily via the angiotensin II Type 1 (AT1) receptor, while AT2 receptor signalling may act as an antifibrotic factor, inhibiting fibroblast proliferation, and matrix synthesis [79, 80]. In obese Zucker rats, inhibition of the angiotensin-converting enzyme (ACE) or blocking the AT1 receptor ameliorates cardiac fibrosis by lowering collagen and TGF-β expression [81]. These findings suggest that TGF-β may be required for AngII to exert fibrogenic activity. In addition to TGF-β signalling, AngII activates multiple other intracellular signalling molecules such as mitogen-­activated protein kinases (MAPKs), as well as increases intracellular reactive oxygen species (ROS) levels in isolated cardiac fibroblast cells, which can be completely blocked by the AT1 antagonists [80, 82]. Despite their beneficial effects *in vitro* and in preclinical models, inhibitors of the angiotensin signalling pathway, such as ACE inhibitors and angiotensin receptor blockers (ARBs), show extremely limited clinical benefit and are not approved for the treatment of cardiac fibrosis.

While the RAAS system is better characterized in the cardiovascular network, its contribution to fibrosis in other tissues in obesity and insulin resistance is less known. In the liver, inhibition of ACE or the AT1 receptor blocker protects the liver from metabolic dysregulation while significantly reducing liver fibrosis in obese Zucker rats [83]. In adipose tissue, overexpression of angiotensinogen and AngII has been shown to accelerate inflammation and fibrosis by inducing macrophage infiltration [84, 85]. Moreover, an increased level of AngII is also associated with skeletal muscle fibrosis, which is evidenced by increased levels of hydroxyproline [86]. These findings suggest that RAAS components may exert a universal fibrogenic activity. However, more researches on their effects outside the cardiovascular system are warranted. The clinical efficacy of RAAS inhibitors against fibrosis merits further investigations.

### TGF-β

TGF-β is an important regulator of the profibrotic response that promotes ECM deposition. In obesity, increased TGF-β signalling has been suggested to exert pro-fibrogenic actions by stimulating the expression of tissue inhibitors of metalloproteases (TIMPs), including TIMP-1, TIMP-3, and TIMP-4, and connective tissue growth factor (CTGF) [55, 87]. In endomyocardial biopsy samples from patients with left ventricular (LV) hypertrophy and HFpEF, increased TGF-β expression leads to interstitial fibrosis which increases cardiomyocyte stiffness and impairs LV relaxation [55]. In adipose tissue of high fat diet-fed obese mice, increased collagen deposition is associated with TGF-β mediated TIMP-1 expression [88]. TGF-β expression is positively correlated with the expression of TIMP-1, TIMP-3, and TIMP-4 in adipose tissue of people with obesity [87]. In addition, TGF-β signalling activation is linked to hepatic steatosis, fibrosis, and insulin resistance in high fat-fed obese mice [89, 90].

TGF-β regulates ECM by direct effects on ECM-producing fibroblast cells. TGF-β signalling has been shown to regulate alpha-smooth muscle actin (α-SMA), a marker of myofibroblast differentiation, CTGF, and collagen Type I in primary cardiac fibroblast cells [55]. Inhibiting the TGF-β signalling pathway prevents myocardial fibrosis in an experimental rat model of hypertension [91]. Moreover, liver-specific overexpression of TGF-β in mice is associated with activation of HSC, evidenced by an increase in matrix proteins such as fibronectin and collagen Types I, III, and IV [92].

The fibrogenic action of TGF-β has been attributed to mechanisms involving canonical small mothers against decapentaplegic (SMAD) signalling [93, 94]. TGF-β binds to receptor kinases to phosphorylate and activate SMAD2 and SMAD3, which form a complex with SMAD4 followed by nuclear translocation and regulation of the expression of target genes [95, 96]. When SMAD3 is deleted in HSC, TGF-β-induced collagen I expression is significantly reduced, whereas overexpression of SMAD2 has the opposite effect [97]. TGF-β also plays a role in the induction and progression of endothelial-to-mesenchymal transition, a process that converts endothelial cells into mesenchymal cells that can then be differentiated into ECM-producing fibroblast cells [98]. This process is mediated through both SMAD signalling as well as the SMAD-independent intracellular signalling such as by c-Abl kinase and protein kinase C-δ [99], which increases the expression of myofibroblast-specific and profibrotic macromolecules including α-SMA, Col I, Col III, TIMP1, and fibronectin [98]. In addition to its profibrotic phenotype in the endothelial cells, TGF-β could execute anti-inflammatory effects in macrophages through phagocytosis of apoptotic cells [100–102]. Macrophages are highly responsive to TGF-β stimulation, mediating fibrotic responses by secreting cytokines, growth factors, and matricellular proteins when polarized to an M1 phenotype [103, 104]. To what extent the profibrotic action of TGF-β is dependent on macrophage-mediated mechanisms is unclear. Regardless, cellular sources of TGF-β include many cell types such as macrophages, lymphocytes, fibroblasts, endothelial cells, and platelets, and TGF-β contributes to tissue fibrosis in a cell type- and context-dependent manner, which was recently reviewed by Frangogiannis *et al.* [104].

### ROS

Obesity is often accompanied by oxidative stress, which is manifested by an imbalance between the generation of ROS and the scavenging capacity of the antioxidant system [105, 106]. Excess ROS generation in response to overnutrition has been implicated in the fibrogenic action of cytokines, AngII, and TGF-β in a variety of cardiovascular diseases including cardiac fibrosis and diastolic dysfunction [107, 108]. NADPH oxidases (NOXs) are membrane-bound enzymes responsible for generating cytosolic ROS. Endothelial cell-specific NOX2 overexpression in AngII-infused mice results in fibroblast activation and an increase in collagen deposition in the heart [109]. An increased level of ROS is also observed in cardiomyocytes and endothelial cells in the heart of patients with HFpEF, accompanied by increased collagen deposition [53, 55]. ROS-mediated reduction of nitric oxide bioavailability to cardiomyocytes causes cardiac hypertrophy and stiffness by lowering protein kinase G activity, which has been linked to hypophosphorylation of the cytoskeleton protein titin [53].

In addition to the heart, elevated ROS levels in adipose tissue of high-fat diet-fed mice are associated with increased collagen deposition as well as decreased adipogenesis and mitochondrial function of the adipocytes [110]. These adverse effects are reversed by a reduction in ROS generation caused by vitamin E supplementation, as reflected by decreased expression of NOX4 and lipoperoxide levels [110]. Moreover, growing evidence suggests that ROS plays an important role in activating HSC and their transdifferentiation into myofibroblast cells, which express myogenic markers such as α-SMA and are required for the initiation of liver fibrosis. HSC activation is found to be highly correlated with oxidative stress in carbon tetrachloride (CCl4)-mediated liver fibrosis and antioxidant activity is inversely related to HSC activation [111]. NOX4-deficient HSCs have significantly lower ROS production and fibrogenic marker expression [112]. It is recently demonstrated that inhibiting NOX1/NOX4 reduces PDGF-induced ROS production and proliferative gene expression in primary mouse HSCs [113].

### Mechanisms by which ECM remodelling in pathological states impacts tissue function

**Figure 3:**
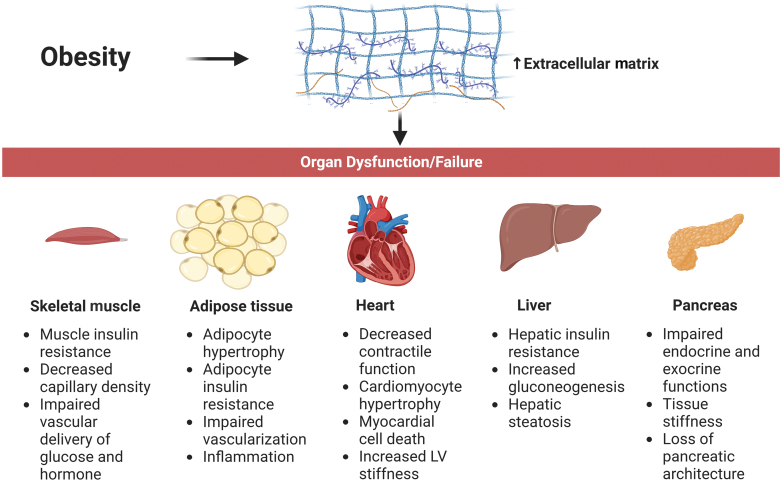
The maladaptive ECM remodelling impairs metabolic tissue function in obesity. ECM remodelling in response to overnutrition contributes to tissue dysfunction in obesity. It affects a wide range of tissues including the skeletal muscle, adipose tissue, heart, liver, and pancreas, all of which are central for controlling fuel metabolism.

Herein, we postulate potential mechanisms whereby mechanical and molecular signals produced from ECM deposition are transduced to affect insulin-sensitive tissues including the skeletal muscle, heart, adipose tissue, liver, and pancreas (Fig. 3). ECM composition is also an important aspect of regenerative medicine as differentiation and proliferation of stem cells are dependent on how extracellular ligands interact with cell surface proteins. This important aspect of ECM-cell interactions is beyond the scope of this review.

### Skeletal muscle

ECM collagen expansion is a hallmark of insulin-resistant skeletal muscle [31, 32]. Recent studies have addressed the role of ECM components (e.g. collagens and hyaluronan) and selected receptors (e.g. integrin and CD44) in contributing to skeletal muscle insulin resistance. In diet-induced obese mice, increased muscle collagen content has been attributed at least partly to an increase in collagen gene expression and decreased muscle matrix metalloproteinase 9 (MMP9) activity [2]. Genetic deletion of MMP9 in mice exhibits increased collagen IV deposition in muscle and exacerbates diet-induced muscle insulin resistance. These effects are accompanied by decreased muscle capillary density [4]. Increased muscle collagen content is believed not only to provide physical barriers to the muscle, resulting in impaired vascular delivery of glucose and hormones, but to transduce intracellular signalling via integrin receptors. Obese mice with global loss of integrin α2β1 are less susceptible to the development of skeletal muscle insulin resistance [2]. It is the fact that increased muscle insulin action in these mice is linked to high levels of collagen expression and improved muscle vascularization, suggesting that integrin α2β1 signalling is essential for collagens to send signals to promote insulin resistance. Furthermore, obese mice with muscle-specific deletion of ILK, a downstream component of the collagen-integrin signalling, improve muscle insulin action [6]. This beneficial effect is accompanied by increased insulin-stimulated AKT phosphorylation and p38 dephosphorylation and improved muscle capillarization. Similarly, data from muscle-specific overexpression of a disintegrin-like and metalloprotease with thrombospondin type 1 motif 9 (ADAMTS9), a secreted MMP, reveal that ADAMTS9 can partially abrogate insulin sensitivity by modulating integrin signalling via increasing ILK and PINCH expression [114].

Pharmacologic and genetic studies suggest that hyaluronan and its receptor CD44 play a role in the aetiology of skeletal muscle insulin resistance *in vivo*. ECM hyaluronan content is increased in the insulin-resistant muscle of obese mice, and intravenous infusion of pegylated human recombinant hyaluronidase PH-20 (PEGPH20) that reduces muscle hyaluronan levels increases muscle glucose uptake during a hyperinsulinemic–euglycemic clamp [3]. This increased muscle insulin action is associated with improved muscle vascularization and increased insulin signalling in muscle. Likewise, genetic deletion of CD44 ameliorates diet-induced skeletal muscle insulin resistance accompanied by improved muscle vascularization [7]. Interestingly, the metabolic beneficial effect of PEGPH20 is dependent upon the presence of CD44 as when PEGPH20 is given to CD44 null mice, its role in improving muscle insulin action in obese mice is absent [7]. CD44 is also linked to Type 2 diabetes and insulin resistance in patients through regulating adipose tissue inflammation which will be discussed later [43, 115]. Taken together, these findings imply that excessive ECM deposition can promote skeletal muscle insulin resistance by activating specific integrin receptors and CD44 signalling. A recent study discovered a unique regulatory route by which insulin initiates slowing and/or termination of its signalling via an integrin αvβ5-dependent pathway [116].

### Heart

Fibrotic alterations in the heart as a result of overnutrition play an important role in the pathophysiology of heart failure, particularly HFpEF, which is the most common kind of heart failure, and the vast majority of those affected are overweight or obese [117]. Studies have revealed elevated collagen deposition in the myocardium of patients with heart failure and metabolic comorbidities such as obesity, diabetes, and hypertension [53, 118]. The relationship between heart failure and comorbidities of obesity and diabetes also extends to insulin resistance, which has independent adverse effects on cardiac function that can be profound [119]. A variety of genetically altered mouse models with perturbed insulin signalling have suggested that disruption of the insulin signalling in the heart causes pathophysiological consequences including decreased contractile function and altered cardiac size either under basal conditions or in the presence of pathological stressors (e.g. myocardial infarction) [120–124]. As a growing body of evidence has suggested a link between muscle ECM remodelling and insulin resistance [2–4], one can speculate that pathological cardiac ECM remodelling underlies insulin resistance in cardiac muscle and may be associated with cardiac dysfunction potentially having an impact in patients with HFpEF and metabolic comorbidities of obesity. Gene expression of fibrotic markers such as *CTGF* and collagen isoforms (*COL1A1*, *COL3A1*, *COL4A1*) is upregulated in the heart tissue of diet-induced obese mice, which also exhibit cardiac insulin resistance [125]. Pirfenidone, an antifibrotic drug with an unknown mechanism of action, inhibits cardiac fibroblast proliferation, myofibroblast differentiation, and migration *in vitro* and reduces the assembly of fibril collagen through attenuation of TGF-β production [126]. Moreover, the antifibrotic effect of pirfenidone in the heart is associated with improved insulin resistance in obese mice [127, 128]. These changes in the ECM environment are also associated with apoptosis, hypertrophy, and impairment of the LV function of the heart [129]. In humans, histological analysis of hearts obtained from patients with HFpEF reveals increased collagen content, collagen cross-linking, and LOX expression. These changes are linked to increased LV stiffness and decreased diastolic function [55]. The amount of insoluble collagen is more significant than the amount of soluble collagen, implying that collagen quality and quantity may have an impact on cardiac function [130].

In addition, alterations in myocardial ECM can lead to stiffening of the ventricles and negatively affect both contraction and relaxation of the heart, contributing to the development of heart failure. Increased cardiac hyaluronan in the heart of hyaluronidase 2 (HYAL2)-deficient animals leads to endothelial-to-mesenchymal transition, mesenchymal cell proliferation, and fibrosis, which are accompanied by considerably increased numbers of vimentin-positive cells [131]. In HYAL2 knockout mice, echocardiography data reveal increased isovolumic relaxation time, indicating diastolic dysfunction. Similarly, interruption of normal hyaluronan catabolism causes cardiac abnormalities in patients with a HYAL2 mutation [132]. Pharmacologic and genetic studies were used to address the impact of fibronectin on heart function. In an experimental mouse model of ischaemia/reperfusion injury, fibronectin inhibition lowers collagen deposition and attenuates adverse cardiac remodelling and infiltration of the myocardium with immune cells [133]. Konstandin *et al*. used a genetic approach to investigate the role of fibronectin in the pressure-overloaded heart, where fibronectin removal reduces cardiomyocyte hypertrophy, delays the onset of heart failure, and increases survival [134]. Taken together, these findings imply that therapeutic strategies that aim at lowering ECM deposition can be used to protect against cardiac dysfunction, especially in individuals with HFpEF.

### Adipose tissue

Adipose tissue undergoes major remodelling during weight gain due to adipocyte hypertrophy and/or hyperplasia. The ability of an adipocyte to expand is dependent on the elasticity of the ECM. Adverse changes in the ECM environment impair ECM flexibility, adipocyte expansion, and tissue plasticity and function. Various models of overnutrition have shown increased ECM deposition in adipose tissue. Increases in isoforms of collagens (e.g. Col I, IV, V, VI, VII, VIII, IX, and XXIV), noncollagen proteins [e.g. secreted protein acidic and rich in cysteine (SPARC), fibronectin, thrombospondin-1, hyaluronan, elastin], and their modifying enzymes (e.g. MMPs, TIMPs, and LOX) have been reported [28, 135, 136]. The decreased capacity for adipocyte expansion and lipid storage due to fibrosis can also impair adipogenesis. This results in accumulation of lipid metabolites, enlarged lipid droplets, and lipotoxicity in tissues that are not well for fat storage [137–139]. These effects are mediated by an integrated response from multiple cell populations including adipocytes, endothelial cells, preadipocytes, adipose stem and precursor cells, fibroblasts, macrophages, pericytes, B cells, T cells, and other immune cells. The cell type-specific contributions to the regulation of adipose tissue function have been extensively reviewed recently by Sun *et al.* [41].

In response to insulin, glucose transporter type 4 (GLUT4) is translocated to the adipocyte cell membrane so that glucose may be consumed for energy storage. It has recently been suggested that abnormal ECM deposition and activation of ECM membrane receptors are important in contributing to adipose tissue insulin resistance [8]. It is worth noting that many extracellular pathways and signals regulate insulin sensitivity and they do so in a tissue-specific manner. For example, SPARC [140], thrombospondin-1 [141], fibronectin [142], elastin [143], MMP14 [144], and endotrophin [145] related pathways have been implicated in the regulation of insulin action. Herein, we narrow the focus to the roles of the collagen-integrin-ILK pathway and hyalurona-CD44 pathway as examples of processes involved in obesity-associated insulin resistance in adipose tissue. Adipocyte-specific deletion of ILK decreases fat mass and improves glucose tolerance in high fat diet-fed obese mice [8]. These mice also display an increase in insulin-stimulated glucose uptake in brown adipose tissue, indicative of activation, and increased thermogenic activity of brown adipose tissue. The anti-lipolytic action of insulin is also improved in the adipocyte ILK-deficient obese mice. These beneficial effects are associated with enhanced vascularization and reduced expression of CD36 in white adipose tissue and increased AKT phosphorylation and p38/JNK dephosphorylation in brown adipose tissue. The greater sensitivity to insulin of ILK-deficient adipocytes suggests that the presence of this highly conserved intracellular protein is necessary for the development of insulin resistance.

Moreover, CD44 is strongly associated with adipose tissue insulin resistance. Kodama *et al*. showed that obese mice had higher adipose tissue CD44 levels compared to lean mice [43]. Global deletion of *CD44* in mice attenuates the development of obesity-induced adipose insulin resistance and glucose intolerance [43]. In addition, antibody neutralization of CD44 reduces obesity-induced adipose tissue inflammation, as demonstrated by decreased expression of immune cell markers (CD68, F4/80, CD3e, and CD19), proinflammatory cytokines (TNF-α, IL-1β, IL-6, and IFN-γ), and monocyte chemoattractant protein-1 (MCP-1) [44]. Increased expression of CD44 in adipose tissue is shown to be associated with inflammation and insulin resistance in patients with Type 2 diabetes, which is consistent with *in vivo* evidence [43].

### Liver

Liver function is compromised in association with hepatic steatosis in patients with obesity. This pathophysiological change promotes hepatic insulin resistance, which results in a diminished capacity to suppress glucose production from the liver after a meal. In the liver, HSCs, portal fibroblasts, and myofibroblasts cells are the major sources of ECM production [146]. These cells have been suggested to have pro-fibrogenic properties in the presence of overnutrition [147, 148]. In obese mice, increased expression of ECM proteins including collagen I, α-SMA, and vimentin is associated with hepatic insulin resistance [149, 150]. Furthermore, the livers of patients with diabetes have worse steatosis and higher perisinusoidal collagen IV, laminin, and α-SMA levels than those in healthy controls [151]. The mechanism by which hepatic ECM deposition leads to insulin resistance is at least partially attributed to integrin signalling. Williams *et al*. found that hepatocytes isolated from high fat diet-fed mice had higher expression of α1β1 collagen-binding integrin than chow diet-fed controls [5]. Paradoxically, integrin α1β1 null mice have higher fasting insulin levels and increased endogenous glucose production during a hyperinsulinemic-euglycemic clamp, indicative of hepatic insulin resistance [5]. These findings suggest that integrin α1β1 protects against diet-induced hepatic insulin resistance, which opposes the role of integrin α2β1 in regulating muscle insulin resistance [2]. Despite being major collagen-binding receptors, integrins α1β1 and α2β1 exert distinct cellular functions, where integrin α1β1 is antifibrotic and proangiogenic and integrin α2β1 is profibrotic and anti-angiogenic [152–155]. However, the exact mechanisms by which the ECM signals through integrin receptors to regulate insulin action remain to be investigated. Moreover, hepatocyte-specific ILK deficiency in mice ameliorates high fat diet-induced hepatic insulin resistance [42]. The insulin sensitizing effect of ILK deletion is also associated with improved hepatic steatosis in obesity [6, 42]. Overall, these studies highlight the significance of the ECM-integrin-ILK signalling in regulating hepatic insulin action and steatosis in obesity.

### Pancreas

The ECM composition is critical to the survival, proliferation, and function of the pancreatic islets. As a major determinant of microcirculatory architecture, the ECM is also critical for nutrient sensing and insulin secretion by regulating islet perfusion. In both humans and rats, the pancreatic ECM is organized as an interstitial matrix and the basement membrane [156]. The former is composed of fibrillar Type I and Type III collagens, Type VI collagen, and fibronectin, and the latter are made up of non-fibrillar collagens, laminins, heparan sulphate proteoglycans, and hyaluronan. Components of the basement membrane, classified as peri-islet and intra-islet ECM, promote adhesion, provide structural support and activate intracellular signalling pathways [157, 158]. Excessive deposition of the ECM in the pancreas, or pancreatic fibrosis, can lead to severe pathological consequences impairing its endocrine as well as exocrine functions [159]. Like the liver, in response to injury or inflammation, quiescent pancreatic stellate cells (PSCs) undergo the transition into activated myofibroblast phenotype, which promotes excessive production of ECM components, resulting in increased tissue stiffness, loss of pancreatic architecture, deformation of ducts, and changes in islet function [160]. Despite the vast amount of evidence implicating pancreatic fibrosis in chronic pancreatitis and pancreatic cancer [159, 161], pathophysiological remodelling of the islet ECM and its functional impact in response to overnutrition and during metabolic diseases are less studied. Excess ECM accumulation around islet blood vessels is a pathological feature of diabetic pancreatic islets [162]. In *db*/*db* mice, increased deposition of ECM components is associated with structural changes in the islet exocrine interface or peri-islet area, indicative of loss of functional communication between the cells [163]. These changes have been associated with the loss of adherent junctions and desmosomes, which promote fibrosis and islet amyloid deposition. It is proposed that increased oxidative stress promotes MMP expression, resulting in impaired cell communication and islet dysfunction such as β cell loss and decreased insulin secretion [164]. Pericytes at the endocrine-exocrine interface of the pancreas have been demonstrated to acquire a myofibroblast-like phenotype that promotes fibrosis by increasing ECM deposition around blood vessels [163, 165]. Immunohistochemical analysis from Type 1 diabetic mice revealed significant accumulation of hyaluronan in both peri-islet and intra-islet ECM [166]. Interestingly, hyaluronan deposition is observed at sites of inflammation, which are identified by clusters of CD45^+^ leukocytes [166]. Yet it is unclear whether pancreatic fibrosis initiates local inflammation or inflammation leads to fibrosis and loss of islet function [167].

## Use of antifibrotics in metabolic diseases

Accumulation of ECM components is increasingly recognized as an important pathogenic process that contributes to insulin resistance and metabolic dysregulation in insulin-sensitive tissues. Therefore, therapies that target pathological ECM remodelling or fibrosis could become an attractive strategy for improving insulin action and its associated cardiometabolic complications of obesity. Preclinical and clinical studies that examined the effects of antifibrotics in metabolic diseases or related conditions are few (Table 1). Pirfenidone and nintedanib are the two antifibrotic therapies that have been approved for the treatment of idiopathic pulmonary fibrosis. Although these drugs have not been tested in obese state, it is shown that pirfenidone has beneficial effects on improving liver fibrosis in rodent models [178]. Pirfenidone has also been shown to abrogate cardiac fibrosis and stiffness and improve LV function in preclinical studies [172–175]. Pirfenidone exerts its antifibrotic action by inhibiting collagen expression, α-SMA expression, and TGF-β mediated transdifferentiation of fibroblast to myofibroblast cells. In the clinic, pirfenidone has been tested in treating patients with chronic hepatitis C and advanced liver fibrosis, both of which exhibit favourable clinical outcomes [178, 179]. Moreover, in a Phase 2 clinical trial (PIROUETTE) among patients with HFpEF and myocardial fibrosis, pirfenidone reduces myocardial extracellular volume despite no significant changes in LV diastolic function [180]. The clinical effectiveness and safety of pirfenidone in HFpEF require further trials.

**Table 1:** Preclinical and clinical use of antifibrotic agents in metabolic disease.

Preclinical studies				
Organ	Antifibrotic agent	Experimental model	Phenotype	References
Liver	Pirfenidone	• *Animal:* male mice (concanavalin A-induced liver fibrosis) • *Dose:* 125 mg/kg/day (2 weeks) • *Administration:* intraperitoneal	• Reduced expression of type II and IV collagens and α-SMA • Decreased serum levels of TGF-β, TNF-α, and TIMP1	[168]
		• *Animal*: male mice (fibrosis induced by CCl4) • *Dose:* 100/300/600 mg/kg (4 or 14 weeks) • *Administration:* in diet	• Reduced collagen deposition at 300 and 600 mg/kg pirfenidone • No effect on inflammation	[169]
		• *Animal*: male cirrhotic Wistar rats (fibrosis induced by CCl4 and bile-duct ligation) • *Dose:* 500 mg/kg per day (3 weeks) • *Administration:* gastric gavage	• Decreased gene expression of collagens I, III, and IV, TGF-β, Smad-7, TIMP-1, and PAI-1 • Reduced activation of HSCs	[170]
	Fluorofenidone	• *Animal*: male albino Wistar rats (pig serum‑induced liver fibrosis) • *Dose:* 240 mg/kg/day (4 weeks) • *Administration:* intragastric route	• Decreased collagens I and III and α-SMA at the mRNA and protein levels	[171]
Heart	Pirfenidone	• *Animal*: male Wistar rats (streptozotocin-induced diabetes) • *Dose:* 200 mg/kg/day (4 weeks) • *Administration:* drinking water (0.2–2 g/L)	• Decreased perivascular and interstitial collagen and attenuated diastolic stiffness	[172]
		• *Animal*: male Sprague-Dawley rats (myocardial infarction model) • *Dose:* 1.2% pirfenidone (4 weeks) • *Administration:* in diet	• Decreased total and non-scar fibrosis • Improved LV function	[173]
		• *Animal*: male Wistar hypertensive rats (deoxycorticosterone acetate-SALT) • *Dose:* 250–300 mg/kg/bw (2 weeks) • *Administration:* in diet	• Normalized collagen deposition and diastolic stiffness	[174]
		• *Animal*: male C57BL/6J mice (pressure-overload induced heart failure) • *Dose:* 400 mg/kg/day (4 weeks) • *Administration:* gastric gavage	• Inhibited TGF-β mediated collagen I expression in fibroblast cells • Prevented TGF-β mediated changes in claudin 5 expression in cardiac fibroblast and endothelial cells • Improved LV systolic function	[175]
Adipose tissue	Isoliquiritigenin	• *Animal*: C57BL/6 mice (high fat diet-induced adipose tissue fibrosis) • *Dose:* 0.5% w/w (20 weeks) • *Administration:* in diet	• Reduced fibrotic area, TNF-α, COL1, and TGF-β1 expression	[88]
Skeletal muscle	Nintedanib	• *Animal*: porcine model (Volumetric muscle loss-induced fibrosis) • *Dose:* 300 mg/day (30 days) • *Administration:* gastric gavage	• Reduced fibrosis and muscle stiffness	[176]
	PEGPH20	• *Animal*: male C57BL/6J (high fat diet-induced skeletal muscle insulin resistance) • *Dose:* 0.1 and 1 mg/kg (24 days) • *Administration:* tail vein	• Reduced hyaluronan in muscle ECM. • Increased insulin signalling and muscle vascularization • Suppressed adipocyte lipolysis and hepatic glucose production	[3]
Pancreas	PEGPH20	• *Animal*: genetically engineered pancreatic cancer mouse model • Dose: once weekly (dose not specified) (3 weeks) • *Administration:* intravenous	• Decreased hyaluronan deposition and reduced interstitial fluid pressure (IFP) • Improveed survival in animals with advanced and metastatic cancer	[177]
*Clinical trials*				
Organ	Antifibrotic agents	Diseases	Study outcomes	References
Liver	Pirfenidone (NCT02161952)	Chronic hepatitis C	• Reduced progression of inflammation, fibrosis, and accumulation of fat in hepatocytes • Enhanced hepatic expression of the anti-fibrogenic receptor CB2 and decreased serum levels of TGF-β1 and IL-6	[178]
	Pirfenidone (PROMETEO; NCT04099407)	Advanced liver fibrosis	• Reduction in fibrosis score • Decreased levels of alanine transaminase (ALT) and/or aspartate aminotransferase (AST), albumin, and serum concentrations of TGF-β, IL-1, and IL-6	[179]
Heart	Pirfenidone (PIROUETTE; NCT02932566)	Heart failure with preserved ejection fraction (HFpEF)	• Decreased myocardial extracellular volume • No change in LV diastolic function	[180]

In preclinical studies, nintedanib is shown to reduce muscle fibrosis and stiffness in a porcine model of volumetric muscle loss-induced fibrosis [176]. Other antifibrotic agents have also emerged from preclinical studies. Flurofenidone, a recently identified water-soluble pyridine, attenuates liver fibrosis by inhibiting HSC activation via the TGF-β/SMAD and MAPK signalling pathways [171]. Supplementation of isoliquiritigenin, a flavonoid from *Glycyrrhiza uralensis*, diminishes adipose tissue fibrosis by suppressing the innate immune responses in high fat diet-fed obese mice [88]. PEGPH20 not only reduces muscle hyaluronan content and improves high fat diet-induced muscle insulin resistance in mice [3], but also decreases hyaluronan deposition in the pancreas and improves survival in animals with advanced and metastatic pancreatic cancer [177]. Despite the beneficial effects of these antifibrotic agents in metabolism, their safety and efficacy in clinical use have not been tested and warrant further investigations. Given our current understanding of the mechanistic links among ECM remodelling, cell surface receptors, and insulin action, therapies that target the ECM membrane receptor signalling such as specific integrin and CD44 signalling may provide novel insights into new therapeutic strategies.

In addition to developing new antifibrotic drugs and repurposing existing therapies for combating fibrosis for the benefit of metabolic diseases, preventive strategies are important to be considered. Amongst the many benefits of a healthy diet and regular physical activity are prevention of hepatic fibrosis and beneficial effects on the cardiovascular system, adipose tissue, and skeletal muscle via organ-crosstalk [181]. Cold exposure induces a fibrogenic-to-adipogenic phenotypic shift in stromal cells, therefore preventing adipose fibrosis from aging [182]. Moreover, anti-inflammatory supplements/diets have been shown to decrease cardiac fibrosis and protect patients from cardiometabolic risks [183]. In the context of non-alcoholic fatty liver disease (NAFLD), a multifaceted approach that combines pharmacological interventions and lifestyle modifications may offer the greatest prospects for effectively managing NAFLD-associated fibrosis and inflammation [184].

## Concluding remarks

Maladaptive ECM remodelling, which ultimately leads to the clinical condition of fibrosis, contributes to obesity-associated insulin resistance and metabolic disorders. It does so at least partially through interacting with cell membrane receptors such as integrins and CD44. Preclinical evidence derived from pharmacological and genetic studies has enhanced our understanding of the underlying mechanisms by which collagen-integrin-ILK and hyaluronan-CD44 signalling pathways regulate insulin action and tissue function in skeletal muscle, liver, and adipose tissue. It is possible that these pathways could also play a key role in modulating cardiac insulin signalling and associated cardiac function, which necessitates further *in vivo* studies. Clinical use of antifibrotic therapies in metabolic diseases may prove to be promising, yet currently available antifibrotics are limited, which narrows their repurposing and general application. Therefore, developing novel approaches against maladaptive ECM remodelling and associated membrane receptor signalling is timely and will benefit from current and evolving knowledge from preclinical and clinical evidence. Complex pathways downstream of integrin receptors such as ILK and other signalling pathways that are parallel to ILK are exciting areas of future research that may be of therapeutic significance.

**Box 1 ILK and CD44.**

**ILK**

ILK is a highly conserved and widely expressed protein that acts as a scaffold for many proteins of the cell adhesome and primary modulator of integrin signalling [185]. It interacts with the cytoplasmic domain of integrins β1, β2, and β3 as well as a variety of cytoskeleton-associated proteins and is a component of the ILK-PINCH-Parvin (IPP) complex [186, 187]. It has been suggested that ILK may regulate intracellular signalling by attracting a kinase or group of kinases to a multiprotein complex, which is consistent with its role as a scaffolding protein. Activation of the pseudokinase domain of ILK promotes the recruitment of adaptor proteins and/or signalling molecules, including insulin-related proteins such as PKB/AKT, PDK1, and GSK-3, which are involved in regulating insulin action [188, 189].

**CD44**

CD44 is a non-kinase transmembrane glycoprotein made up of extracellular domains, a membrane-proximal region, a transmembrane domain, and a cytoplasmic tail [190]. It is a single-chain molecule that is encoded by a single gene on Chromosome 11 in humans and Chromosome 2 in mice [191]. CD44 is expressed by most cells and interacts with a variety of ligands, including hyaluronan, osteopontin, and chondroitin [192, 193]. Hyaluronan, the most specific ligand for CD44, activates the CD44 signalling pathway by inducing conformational changes favouring adaptor protein recruitment to the CD44 cytoplasmic tail [194]. Activation of the CD44 pathway has been linked to a number of biological processes, including development, cancer metastasis, and cell adhesion [194, 195]. It also plays a role in the production of proinflammatory cytokines as well as macrophage and neutrophil migration [196]. The *CD44* gene has been linked to the molecular pathogenesis of Type 2 diabetes in humans [43] and its role in metabolism has been recently reviewed [197].
